# Supplementary material for: Patterns of Intron Gain and Loss in Fungi
Source: PLoS Biol. 2004 Nov 30;2(12):e422. doi: 10.1371/journal.pbio.0020422 (PMC532390; doi:10.1371/journal.pbio.0020422)
Supplement: Table S1 — Also available at http://genes.mit.edu/NielsenEtAl/. (4.3 MB ZIP). [file pbio.0020422.st001.zip › NielsenEtAl/html/139.html]

AN4884.1.NCU02270.1.MG00918.1.FG06301.1


```
 CLUSTAL W (1.82) Multiple Sequence Alignments - Introns Inserted


Sequence 1: NCU02270.1	782 aa
Sequence 2: MG00918.1	790 aa
Sequence 3: FG06301.1	787 aa
Sequence 4: AN4884.1	727 aa
Alignment Length: 833 aa
Number Identitical Residues: 180 aa
Alignment Score (without introns) 12781


MG00918.1 	-------MEDPLQETRVYDFIEDKLQSTTDLDHLDSLLASVEHQRTQLQTQLDDATRELD
NCU02270.1	-------MEF---DIRVEDYLDDKLQSTTDFDHLDTLLNSVEHQRSQLQSQLDNATKELE
FG06301.1 	MATTAAHPQGTSLDIRVEDFLDDKLQSTADLENLDTLLENVELQRNQLQSQLDTAVKELE
AN4884.1  	------------------------------------------------------------
          	                                                            

MG00918.1 	AARRSGGDRQEALARQIDDFEQLQRSIDVRLQ~IVSASDAPDEAIRRLEPPMHQLHRVKL
NCU02270.1	EARRSSAERQAALAAQIDEFQKLQQSIDVRLK~IVAASDAPDEAIRRLEQPMKQLHKVDL
FG06301.1 	ETRRTADDRQASLTARIQEFQKLQESIDLRAQ~IYAASDAPDQAIERLKSPMNKIKAVER
AN4884.1  	---------------------------MSRPP0TLTGSNASDEAAKRFEASIKKLRRLEL
          	                             *     :.*:*.*:* .*:: .::::: :. 

MG00918.1 	ARDYLQLLHDVEALR~REARDHLPADPKAALLPYSRLKQLSSRLRELQGTADGAATHLVA
NCU02270.1	AHRYLLLLQHVQELR~NAARAHLPQSPKAALEPYTELKQLSLRLRELQGAADGAAVHLVA
FG06301.1 	AQSYLVLLQDAEKLR~AEAKSFLPQDPKAALEPYIKLKQHVLKLQGLPGQEG---LHLVA
AN4884.1  	SQSYVALLKDAEELS2KEALRNIKSEARLAIASYTQLRNIVESLDKAQPAAEGASPHLVD
          	:: *: **:..: *    *   :  ..: *: .* .*::    *        .:: *** 

MG00918.1 	HVAGVTDVLWDEMKQIMSDEMKALLAARRWPA-EVQPDLVVDEEWRGCFEKLLDLQVP~E
NCU02270.1	HVESVAEALWDEMKKTMWSELEVLLKKRGWP--NVDPESEVDDEWIQCFEKLVDLQVP~E
FG06301.1 	YVENLTRSLWDDIKSTMSGELEEVLKQRKWP--RVEADLQMDEEWINCIEKLIDLQKP~E
AN4884.1  	YVGQMASRLREQMKMEFTNRLRSTLEQMNWPSKELNFPENLRNQWADNVELLLDLQIP2E
          	:*  ::  * :::*  : ..:.  *    **:..::    : ::*   .* *:*** * *

MG00918.1 	VLYSPPDAVLPLLP---IDVMAQIWVKEFRFHFLSDGRPTSNNRLI~GQHCFPWVIRLVS
NCU02270.1	VLYSP--TLVTLLP---MDVMAQIFVKEFRFHFLSD-KPTSNPQGI~NAHCFPWFLSLLD
FG06301.1 	IAHTD--KLVTLLP---FEIMASIFVSEFRFHFLSD-KPTSSPQAF~GTHCLSWFIALVE
AN4884.1  	LETHDSASQKIVQPPVLFALEAMVHPLELRFKYHFSGDKPTNRLDK0PEYFLAHTTDLIN
          	:    .     : *.  : : * :   *:**::  ..  .:.       : :.    *:.

MG00918.1 	TWEDFFRENFGHSLASRFIETSVSSKMIYLDPVCALITSLLPV~LREKIMAAAEEAVLDP
NCU02270.1	KWEIFFRENFAPVLAAKFGQTEVSNKMVYMDPGCAFIASMLPV~MREKVNVTMAEAVKDP
FG06301.1 	KWEDFFRDNLGYLLAEKFRDTDVATNLAYIDPVSALITSLLPM~LKEKTSLVALEAVKSP
AN4884.1  	NFGAFFTSYLQPIFNQKSKTVGDQLAWHFLSASDAYITAILPM0ISA-----------HP
          	.:  ** . :   :  :   .       ::..  * *:::**: :              *

MG00918.1 	SFLSSFMTQLMGFDDELRTRFGYD-GGGGDSDWDGLTAEVLN--LHFAKWLQAEKDFALE
NCU02270.1	AFLSSLLGQLMTFDDNIRSGYSYD-GGDAENGWEGLTGEVLN--THFRTWLEAEKKFALE
FG06301.1 	SFLSSFMSQLMAFDDNVRYKFSYD-SGDAENGWSGLTAYVLD--DHFDTWFKAEKEFALE
AN4884.1  	QLLSHFVHELMDYDNGIRETWNYLPYPYSQEDWRGLTWEVLSKEGWFDRWLQVEKDFALA
          	 :** :: :** :*: :*  :.* .   .:..* ***  **...  *  *::.**.*** 

MG00918.1 	RFEGIMDSADARKIDYDYGGPGKTKPTFGAVRVSDLLRSVTSQYERVRRFSHKLRFLIDI
NCU02270.1	RFNGIMTSSDARKIDYDFAVVGKTKPTFAAVRVADLLRSVTTQYERLRRFSHKLRFLIDI
FG06301.1 	RYNTIIDSQDARNIDYDYALQGKMKPTYAAVRVTDLLRSVTSQYERVRTFKHKIRFLIGI
AN4884.1  	RYEEIVDTPDSGHLDYDGVEPTATKPTRAAIRVNDLLETITERYQPLSSFSQKLRFLIDI
          	*:: *: : *: ::***       *** .*:** ***.::* :*: :  *.:*:****.*

MG00918.1 	QVAILDDFHDRLRGSLEFYYSLTSTVGRTLHGATAQQLASLEGTGALETLCKVYGSADHI
NCU02270.1	QLAILDDYHDHLKGTLEAYQTMTSTVGRAFVGVTKEQLAALEGTGALETLCKVYGSADHI
FG06301.1 	QLEILDAYHDRLRDSLQAYQSMSTTFGRTLA-ANKEDLAVLEGTGGFEVLCKVIGSADHV
AN4884.1  	QITLFDRFHERLHSALEAYLAMTSTIGRTVQGADGQ--ASVEGIAGLERLCRVFGSAEYL
          	*: ::* :*::*:.:*: * ::::*.**:. ..  :  * :** ..:* **:* ***:::

MG00918.1 	ANVLEDWSNEE0FFVVFWEELQARAKESEDQ-ENLAGGLSYENVKDKTSASIG-------
NCU02270.1	INALHDWSNEE0FFVTLWEQLQTRAKANEDQ-SNLAGGMSYDHVKDRISSVVG-------
FG06301.1 	VNTLKDWSNEE~FFVSLWDELQTRALHRASQGNNITSTMSYDDVKDRTSAAVG-------
AN4884.1  	EKKMDDWSNEV~FFVELWSELQERIKQNKHSGKNVAGTMSVADVASRTSSAINNTSGEIS
          	 : :.*****  *** :*.:** *      ...*::. :*  .* .: *: :..::.. :

MG00918.1 	-DDGDGGVIFDETIAAYSARRKAARDFLVSALVDSHQEAFRPYLKRTQWTTISDDPVQA~
NCU02270.1	-TEDDTGVLFDETISAYTMRRKRAQEFLTQVLIQNQKKAFAAYLKRPQWSTISDEPTSG~
FG06301.1 	-EKREDGALFDETATAYSMRRTAAQELLVGALVESHNKAFRAYTTRVQWTTVGETTISD1
AN4884.1  	QDETPEGALFDETASAYRRLRQRSESIIISTLVSNVRSALKSYSRASNWSTISGHSVPA~
          	. .   *.:**** :**   *  :..::  .*:.. ..*: .*    :*:*:.  .    

MG00918.1 	DP-YQLPITAELDEPLR0ILKRNLEFLNRALSTAAFRRVWRDALDKLDETLWHDVLTRHS
NCU02270.1	EAYYQLAPTAELGEPLA0VLKRDLDFLSHALGTAVFRRIWREALEELNNMLWSDVLMANK
FG06301.1 	EL----AITPELDEPLR~ILQRNFDFLVKALSTAVFRRVWREALTQLQEHLWQTVLMRQS
AN4884.1  	TG--HLPPSADLAQTMR~ALSTSITFLSRALGVAPLRRIVRQVLLSIQTYIWSNVLLRNT
          	      . :.:* :.:   *. .: ** :**..* :**: *:.* .::  :*  **  :.

MG00918.1 	FTAFGAAQFMRDLHAVVALVERYIP-----EGSGVALARVQEGVRLLNLPMEADASG---
NCU02270.1	FAASGAAQFRRDVDAISSVVERYIP-----DGS-IALESIKEALHLLNLPVEAPEE----
FG06301.1 	FTTYGATQFCRDGVALVSIVERYIP-----NGS-SILDSLTEGIQLLSLPVEAGEA----
AN4884.1  	FSASGAAQLASDMDHLYSVVDAALGHAVQAGGSKFILQKLNEGLLILNLEAQTPVAEGED
          	*:: **:*:  *   : ::*:  :  : .: **   *  : *.: :*.*  ::    ...

MG00918.1 	-------KAAAVTLRQASDRVFKDNEEAKRLLAELGIDLLSPTNARHILQRRVEMSE---
NCU02270.1	-------NSNTMTLKNAVDRVFTDNTEAKKVLEELEIESLSPPHARQILQRRVENAE---
FG06301.1 	---------GGLTLKEASDRVFTDNEEARKVLEELHLEELSPQSARNILQRRVENNENVG
AN4884.1  	KAPAESAAEKGLGLWEVEKRLFKDNESARETLAELGVETLTESEARSILEKRVEIGS---
          	.:.:.::    : * :. .*:*.** .*:. * ** :: *:   ** **::***  .   

MG00918.1 	-
NCU02270.1	-
FG06301.1 	W
AN4884.1  	-
          	
```
